# Triglyceride–glucose–alanine aminotransferase index: A noninvasive serum predictor for identifying the severity of pediatric nonalcoholic fatty liver disease

**DOI:** 10.1097/MD.0000000000038241

**Published:** 2024-06-28

**Authors:** Eu-Seon Noh, Il Tae Hwang

**Affiliations:** a Department of Pediatrics, Kangdong Sacred Heart Hospital, Hallym University College of Medicine, Seoul, Republic of Korea.

**Keywords:** alanine aminotransferase index, nonalcoholic fatty liver disease, nonalcoholic steatohepatitis, triglyceride–glucose, triglyceride–glucose–alanine aminotransferase index, ultrasound fatty liver index

## Abstract

We hypothesized that the triglyceride–glucose (TyG)–alanine aminotransferase (ALT) index, which combines the TyG index with ALT, may enhance sensitivity and specificity in detecting the severity of nonalcoholic fatty liver disease (NAFLD). A total of 131 NAFLD patients with a mean age of 11.5 ± 2.29 years were enrolled, and severity was assessed by ultrasound fatty liver index (US-FLI) scoring. The TyG–ALT index was defined as ln(fasting triglyceride [mg/dL] × fasting glucose [mg/dL] × ALT [IU/L]/2). Multiple linear regression analysis revealed a significant association between the TyG–ALT index and US-FLI (*β* = 0.317, *P* < .001) after controlling for sex, age, and body mass index. The TyG–ALT index showed a more stable and superior ability to detect the severity of NAFLD compared to both ALT and the TyG index. The area under the curve values, listed in the order of ALT, TyG index, and TyG–ALT index, were as follows: 0.737 (*P* < .001), 0.599 (*P* = .055), and 0.704 (*P* < .001) at US-FLI ≥ 4 points; 0.717 (*P* < .001), 0.720 (*P* < .001), and 0.775 (*P* < .001) at US-FLI ≥ 5 points; and 0.689 (*P* < .05), 0.748 (*P* < .01), and 0.775 (*P* < .001) at US-FLI ≥ 6 points. The TyG–ALT index is associated with US-FLI score and superior to both ALT and the TyG index in predicting NAFLD severity. These findings indicate the potential of the TyG–ALT index in the management of pediatric NAFLD progression.

## 1. Introduction

Nonalcoholic fatty liver disease (NAFLD) is defined as chronic hepatic steatosis in the absence of other causes for secondary fat accumulation in the liver. NAFLD represents a spectrum of liver conditions similar to those caused by alcohol, such as simple steatosis, with progression to nonalcoholic steatohepatitis (NASH) and potential escalation to fibrosis/cirrhosis and hepatocellular carcinoma.^[1]^ The incidence of NAFLD among children has been increasing worldwide.^[2–5]^ Obesity is a major cause of NAFLD as well as dyslipidemia, type 2 diabetes mellitus (T2DM), and metabolic syndrome (MetS) in children and adolescents.^[6–8]^ The increasing prevalence of obesity has recently become a public health concern.

Pediatric NAFLD can be more serious than adult NAFLD; therefore, early screening is important to prevent disease progression.^[9,10]^ Although the gold standard for diagnosing and staging NAFLD is liver biopsy, it is difficult to perform liver biopsy in clinics owing to its invasive nature.^[11]^ Therefore, noninvasive alternatives, such as magnetic resonance imaging and the measurement of biomarkers that can represent liver biopsy results, have been developed recently.^[12]^ Research focusing on new markers to non-invasively identify the severity of pediatric NAFLD has been deemed crucial.

The main pathophysiology of NAFLD is insulin resistance (IR), and studies have already been conducted on noninvasive biomarkers of IR.^[13–16]^ The triglyceride–glucose (TyG) index has emerged as a reliable surrogate marker for IR.^[17,18]^ Alanine aminotransferase (ALT) is used as a standard indicator of liver function to reflect liver inflammation and damage in patients with various chronic liver diseases, including NAFLD. Although ALT can help detect NAFLD, it alone may not be an ideal biomarker. We hypothesized that the combination of TyG, a reliable IR surrogate marker, and ALT, which reflects hepatic inflammation and injury, would increase both sensitivity and specificity for the evaluation of NAFLD. Therefore, this study evaluated the usefulness of the TyG–ALT index, a novel modified TyG index that combines ALT with the conventional TyG index, for the detection of NAFLD and the ability to determine the severity of NAFLD and compared it to both the TyG index and ALT.

## 2. Materials and methods

### 2.1. Study population

This study was a cross-sectional study that analyzed the clinical data of subjects diagnosed with NAFLD at a pediatric endocrine clinic from January 2021 to October 2022. A total of 131 children and adolescents aged 6 to 18 years with a diagnosis of NAFLD according to the criteria presented below were enrolled in this study. This study was approved by the institutional review board (IRB no. KANGDONG 2021-12-007) of Hallym University Gangdong Sacred Heart Hospital.

ALT was used as the initial screening test for NAFLD in children and adolescents who visited the hospital for health checkups. In this study, the cutoff values suggesting liver disease in children and adolescents were based on an ALT level greater than the 97th percentile and was 26 U/L for boys or 22 U/L for girls as presented in the Screening ALT for Elevation in Today’s Youth (SAFETY) study. Ultrasound fatty liver index (US-FLI) scoring was performed for children who met these criteria. The US-FLI is a scoring system ranging from 2 to 8 points based on the intensity of liver/kidney contrast, posterior attenuation of an ultrasound beam, vessel blurring, difficulty visualizing the gallbladder wall, difficulty visualizing the diaphragm, and areas of focal sparing. NAFLD is diagnosed by a minimum score of ≥2 points. The optimal cutoff points for the US-FLI score for predicting NASH in adults and children were ≥4 points and ≥6 points, respectively. Children and adolescents who were treated for viral hepatitis, Wilson disease, and autoimmune hepatitis were excluded. NAFLD subjects with all fasting results, including serum glucose, aspartate transaminase (AST), ALT, total cholesterol (TC), triglycerides (TGs), high-density lipoprotein cholesterol (HDL-C), and insulin, were included in the study.

### 2.2. Measurements

Height was measured using a Harpenden stadiometer (Cat# 98.602VR; Holtain Ltd., Crymych, UK) to the nearest 0.1 cm. Weight was measured using a digital scale to the nearest 0.1 kg. The body mass index (BMI) of each participant was calculated as weight divided by height squared (kg/m^2^). Based on the 2017 Korean national reference, standard deviation score (SDS) values for height, weight, and BMI were assessed using the LMS (L, lambda for the Box–Cox power for skewness; M, mu for the median; S, sigma for the generalized coefficient of variation) method (SDS = [measured value/M]^1/L^/LS).^[19]^ Venous blood samples were obtained after participants fasted for ≥8 h, and biochemical tests, including those for glucose, AST, ALT, TC, TG, and HDL-C, were performed using an automatic analyzer (Hitachi 7600; Hitachi, Tokyo, Japan). Serum insulin levels were measured using a Wizard 1470 gamma counter (Perkin–Elmer, Waltham). Low-density lipoprotein cholesterol (LDL-C) levels (mg/dL) were calculated using Friedewald equation.^[20]^ The Homeostatic Model Assessment of Insulin Resistance (HOMA-IR), TyG index, and TyG–ALT index were calculated as follows: HOMA-IR = (fasting insulin [mIU/L] × fasting glucose [mg/dL]/405),^[21]^ TyG index = ln(fasting TG [mg/dL] × fasting glucose [mg/dL]/2)^[21]^ and TyG–ALT index = ln(fasting TG [mg/dL] × fasting glucose [mg/dL] × ALT [IU/L]/2).

### 2.3. Statistical analysis

The Statistical Package for the Social Sciences version 26.0 (IBM Corp., Armonk) was used to perform the statistical analyses. To determine statistical significance between groups, Student *t* test or the Mann–Whitney *U* test was used for continuous variables. Pearson correlation tests were performed to examine correlations between the TyG–ALT index and clinical parameters. Multiple linear regression analysis was performed to investigate the association between clinical variables (sex, age, BMI SDS, insulin, AST, ALT, TC, HDL-C, LDL-C, non–HDL-C, and US-FLI) and IR surrogate markers (TyG index and TyG–ALT index) after adjusting for sex, age, and BMI SDS. We verified the ability of IR surrogate markers to detect the severity of NAFLD using receiver operating characteristic curves and compared the ability of the TyG–ALT index to detect each stage of NAFLD severity (US-FLI ≥ 4, ≥5, or ≥6 points) to that of ALT and the TyG index. Continuous variables and clinical variables are reported as mean ± SD values and statistical significance was set at *P* < .05.

## 3. Results

The clinical characteristics of the reference population are presented as mean ± SD values (Table 1). Among the 131 participants, 95 were boys and 36 were girls. Pubertal stage, BMI, BMI SDS, glucose, AST, ALT, TC, LDL-C, HDL-C, non–HDL-C, TG levels, insulin, TyG index, TyG–ALT index, and US-FLI score were not significantly different between boys and girls, but age was significantly different (*P* < .001).

**Table 1:** Descriptive characteristics of patients with NAFLD.

	Total (n = 131)	Boys (n = 89)	Girls (n = 42)	*P*
Patient characteristic				
Age, yr	11.43 ± 2.35	11.94 ± 2.13	10.28 ± 2.45	<.001
Height, cm	151.13 ± 13.29	153.96 ± 12.92	144.73 ± 11.92	<.001
Height SDS	0.81 ± 1.21	0.77 ± 1.26	0.93 ± 1.09	.484
Weight, kg	62.17 ± 20.07	65.44 ± 19.89	54.79 ± 18.68	.004
Weight SDS	2.12 ± 1.21	2.10 ± 1.23	2.17 ± 1.17	.763
BMI, kg/m^2^	26.47 ± 4.71	26.95 ± 4.56	25.40 ± 4.92	.076
BMI SDS	2.34 ± 1.13	2.32 ± 1.12	2.39 ± 1.16	.712
Biochemical profile				
Glucose, mg/dL	100.57 ± 24.66	101.91 ± 28.21	96.91 ± 9.25	.321
Uric acid, mg/dL	6.27 ± 1.51	6.44 ± 1.60	5.84 ± 1.16	.043
AST, IU/L	52.51 ± 30.55	49.40 ± 29.65	59.55 ± 31.73	.073
ALT, IU/L	82.23 ± 56.64	79.82 ± 51.35	90.95 ± 67.12	.291
TC, mg/dL	180.02 ± 57.13	177.72 ± 64.86	185.71 ± 30.53	.469
LDL-C, mg/dL	108.94 ± 23.96	106.52 ± 23.80	116.38 ± 23.3	.054
HDL-C, mg/dL	49.58 ± 12.78	49.73 ± 13.29	49.16 ± 11.42	.830
Triglycerides, mg/dL	129.29 ± 74.92	125.34 ± 82.07	141.03 ± 46.98	.323
Non-HDL-C, mg/dL	132.06 ± 58.43	128.80 ± 65.41	140.43 ± 33.93	.320
Insulin, μU/mL	15.87 ± 10.44	15.09 ± 8.94	18.67 ± 14.58	.306
HOMA-IR	3.99 ± 2.87	3.87 ± 2.73	4.47 ± 3.40	.487
TyG index	8.63 ± 0.57	8.59 ± 0.62	8.77 ± 0.37	.128
TyG–ALT index	12.82 ± 1.03	12.78 ± 1.06	12.94 ± 0.94	.431
Imaging characteristic				
US-FLI	3.75 ± 1.53	3.80 ± 1.53	3.64 ± 1.53	.581

ALT = alanine aminotransferase, AST = aspartate transaminase, BMI = body mass index, HDL-C = high-density lipoprotein cholesterol, HOMA-IR = Homeostatic Model Assessment of Insulin Resistance, LDL-C = low-density lipoprotein cholesterol, NAFLD = nonalcoholic fatty liver disease, SDS = standard deviation score, TC = total cholesterol, TyG index = triglyceride–glucose index, TyG–ALT index = triglyceride–glucose–alanine aminotransferase index, US-FLI = ultrasonographic fatty liver index.

In Pearson correlation analysis (Table 2), BMI (*R* = 0.142, *P* = .038), glucose (*R* = 0.329, *P* < .001), AST (*R* = 0.674, *P* < .001), ALT (*R* = 0.749, *P* < .001), TG (*R* = 0.701, *P* < .001), non–HDL-C (*R* = 0.237, *P* = .012), insulin (*R* = 0.263, *P* = .012), HOMA-IR (*R* = 0.388, *P* < .001), TyG index (*R* = 0.793, *P* < .001), and US-FLI score (*R* = 0.414, *P* < .001) were statistically correlated with TyG–ALT index.

**Table 2:** The correlation between the TyG–ALT index and clinical parameters.

	*r*	*P*
Patient characteristic		
Age	0.055	.566
Sex	0.116	.223
Height	0.106	.266
Height SDS	0.030	.750
Weight	0.168	.077
Weight SDS	0.142	.135
BMI	0.197	.038
BMI SDS	0.179	.059
Biochemical profile		
Glucose	0.329	<.001
Uric acid	0.047	.626
AST	0.674	<.001
ALT	0.749	<.001
TC	0.192	.043
LDL-C	0.180	.059
HDL-C	−0.095	.320
Triglycerides	0.701	<.001
Non–HDL-C	0.237	.012
Insulin	0.263	.012
HOMA-IR	0.388	<.001
TyG index	0.793	<.001
Imaging characteristic		
US-FLI	0.414	<.001

ALT = alanine aminotransferase, AST = aspartate transaminase, BMI = body mass index, HDL-C = high-density lipoprotein cholesterol, HOMA-IR = Homeostatic Model Assessment of Insulin Resistance, LDL-C = low-density lipoprotein cholesterol, SDS = standard deviation score, TC = total cholesterol, TyG index = triglyceride–glucose index, TyG–ALT  = triglyceride–glucose–alanine aminotransferase, US-FLI = ultrasonographic fatty liver index.

The results of multiple linear regression analysis for the association between clinical variables and the TyG index or TyG–ALT index are presented in Table 3. Considering TyG index as the dependent variable and sex, age, BMI SDS, insulin, AST, ALT, TC, HDL-C, LDL-C, non–HDL-C, and US-FLI as the independent variables, 24.2% of the variance in TyG index was explained by non–HDL-C (*β* = 0.331, *P* = .001), insulin (*β* = 0.235, *P* = .013), and US-FLI score (*β* = 0.288, *P* = .003). Considering the TyG–ALT index as the dependent variable and sex, age, BMI SDS, insulin, AST, TC, HDL-C, LDL-C, non–HDL-C, and US-FLI as the independent variables, 55.4% of the variance in TyG–ALT index was explained by AST (*β* = 0.554, *P* < .001), non–HDL-C (*β* = 0.168, *P* = .021), insulin (*β* = 0.159, *P* = .029), and US-FLI score (*β* = 0.317, *P* < .001).

**Table 3:** Clinical variables analyzed with the TyG index and TyG–ALT index using multiple linear regression.

	TyG index*			TyG–ALT index†	
	Adjusted *R*^2^ 0.242, *P* < .001			Adjusted *R*^2^ 0.554, *P* < .001	
	Standardized coefficients	*P*		Standardized coefficients	*P*
Non-HDL-C	0.331	.001	AST	0.554	<.001
Insulin	0.235	.013	Non-HDL-C	0.168	.021
US-FLI	0.288	.003	Insulin	0.159	.029
			US-FLI	0.317	<.001

AST = aspartate transaminase, BMI = body mass index, HDL-C = high-density lipoprotein cholesterol, SDS = standard deviation score, TyG = triglyceride–glucose, TyG–ALT index = triglyceride–glucose–alanine aminotransferase index, US-FLI = ultrasonographic fatty liver index.

* Multiple linear regression analysis was performed using sex, age, BMI SDS, insulin, AST, ALT, total cholesterol, HDL-C, low-density lipoprotein cholesterol, non–HDL-C, and US-FLI.

† Multiple linear regression analysis was performed using sex, age, BMI SDS, insulin, AST, total cholesterol, HDL-C, low-density lipoprotein cholesterol, non–HDL-C, and US-FLI.

To find independent risk factors associated with NASH in patients with NAFLD, multiple logistic analysis was performed using confounding factors (Table 4). In model 1, analysis was performed using sex, age, BMI SDS, insulin, AST, TC, HDL-C, LDL-C, non–HDL-C, and TyG index. BMI SDS and TyG index were independent risk factors for NASH, with odds ratios of 3.46 (95% confidence interval [CI], 1.47–8.15; *P* = .005) and 16.22 (95% CI, 2.85–92.36; *P* = .002), respectively. In model 2, analysis was performed using sex, age, BMI SDS, insulin, AST, TC, HDL-C, LDL-C, non–HDL-C, and TyG–ALT index. Here, both BMI SDS and the TyG–ALT index were independent risk factors for NASH, with odds ratios of 1.37 (95% CI, 1.104–1.701; *P* = .004) and 11.78 (95% CI, 2.518–55.091; *P* = .002), respectively.

**Table 4:** Independent risk factors associated with NASH and clinical variables using multiple logistic analysis in patients with NAFLD.

	Model 1			Model 2	
	OR (95% CI)	*P*		OR (95% CI)	*P*
BMI SDS	3.46 (1.47–8.15)	.005	Age	1.36 (0.97–1.89)	.071
Insulin	0.92 (0.84–1.01)	.079	BMI SDS	2.76 (1.06–7.13)	.037
TyG index	16.22 (2.85–92.36)	.002	AST	0.97 (0.93–1.01)	.178
			Insulin	0.92 (0.82–1.03)	.135
			TyG–ALT index	10.36 (2.21–48.51)	.003

Model 1 = Analysis was performed using sex, age, BMI SDS, insulin, AST, total cholesterol, HDL-C, low-density lipoprotein cholesterol, non–HDL-C, and TyG index.

Model 2 = Analysis was performed using sex, age, BMI SDS, insulin, AST, total cholesterol, HDL-C, low-density lipoprotein cholesterol, non–HDL-C, and TyG–ALT index.

AST = aspartate transaminase, BMI = body mass index, CI = confidence interval, HDL-C = high-density lipoprotein cholesterol, NASH = nonalcoholic steatohepatitis, NAFLD = nonalcoholic fatty liver disease, OR = odds ratio, SDS = standard deviation score, TyG index = triglyceride–glucose index, TyG–ALT index = triglyceride–glucose–alanine aminotransferase index.

The areas under the receiver operating characteristic curves (AUCs) for ALT, the TyG index, and the TyG–ALT index are shown in Table 5. The AUC of ALT was 0.737 (95% CI, 0.637–0.837; *P* < .001) at US-FLI ≥ 4 points, showing a good detection ability. However, the AUC of ALT decreased with increasing severity of NAFLD, from 0.717 (95% CI, 0.624–0.810; *P* < .001) at US-FLI ≥ 5 points to 0.689 (95% CI, 0.558–0.689; *P* < .05) at US-FLI ≥ 6 points. The AUC of the TyG index was 0.599 (95% CI, 0.558–0.689; *P* = .055) at US-FLI ≥ 4 points, which did not show a detection ability, but the detection ability improved significantly as the severity of NAFLD increased: AUCs were 0.720 (95% CI, 0.626–0.814; *P* < .001) at US-FLI ≥ 5 points and 0.748 (95% CI, 0.616–0.880; *P* < .01) at US-FLI ≥ 6 points. The TyG–ALT index showed a consistently good detection ability at all severity levels of NAFLD, as follows 0.704 (95% CI, 0.602–0.806; *P* < .001) at US-FLI ≥ 4 points, 0.775 (95% CI, 0.689–0.861; *P* < .001) at US-FLI ≥ 5 points, and 0.775 (95% CI, 0.648–0.902; *P* < .001) at US-FLI ≥ 6 points.

**Table 5:** The ability of ALT, TyG index, and TyG–ALT index to detect the severity of NAFLD according to the US-FLI score using receiver operating characteristic analysis.

	US-FLI ≥ 4 points		US-FLI ≥ 5 points		US-FLI ≥ 6 points	
	AUC (95% CI)	Cutoff values (sensitivity/specificity, %)	AUC (95% CI)	Cutoff values (sensitivity/specificity, %)	AUC (95% CI)	Cutoff values (sensitivity/specificity, %)
ALT	0.737*** (0.637–0.837)	50 (85/59)	0.717*** (0.624–0.810)	48 (97/41)	0.689* (0.558–0.821)	58.5 (87/46)
TyG index	0.599 (0.491–0.708)	8.59 (61/61)	0.720*** (0.626–0.814)	8.62 (80/60)	0.748** (0.616–0.880)	8.81 (80/68)
TyG–ALT index	0.704*** (0.602–0.806)	12.51 (77/57)	0.775*** (0.689–0.861)	12.83 (86/61)	0.775*** (0.648–0.902)	13.18 (80/73)

ALT = alanine transaminase, AUC = area under the receiver operating characteristic curve, CI = confidence interval, NAFLD = nonalcoholic fatty liver disease, ROC = receiver operative characteristic, TyG index = triglyceride–glucose index, TyG–ALT = triglyceride–glucose–alanine aminotransferase, US-FLI = ultrasonographic fatty liver index.

* *P* > .05.

** *P* > .01.

*** *P* > .001.

## 4. Discussion

In the present study, the main findings were that the TyG–ALT index showed a role as a surrogate marker for IR in NAFLD and had a significant association with US-FLI after adjusting for sex, age, and BMI, and TyG–ALT was an independent risk factor for NASH risk in NAFLD patients and superior to both ALT and the TyG index for predicting the severity of NAFLD.

NAFLD is a liver manifestation of MetS, which is associated with IR.^[22,23]^ Research on the detection of NAFLD using IR surrogate markers is ongoing. It is a noninvasive test and, therefore, expected to be used in clinical practice as an alternative to liver biopsy.^[14,15,24,25]^ The gold standard for measuring IR is the hyperinsulinemic–euglycemic clamp. However, it is time-consuming and expensive, which limits its clinical use^[26]^ The TyG index can be estimated clinically from a single blood sample. In addition, they are well-studied surrogate markers in terms of their usefulness in IR. The TyG index, using fasting TG and glucose levels, is emerging as a reliable and simple surrogate marker of IR.^[18,27]^ The TyG index is useful for predicting T2DM, MetS, and NAFLD, which share a common pathophysiology of IR. The role of the TyG index in the mechanism underlying the occurrence of NAFLD is unclear. However, it can be explained as follows. The pathophysiological mechanisms underlying NAFLD involve IR, leading to hypertriglyceridemia and hyperglycemia.^[28,29]^ Abnormal lipid metabolism occurs due to an increased flux of free fatty acids from adipose tissue to non-adipose organs, resulting in TG accumulation and contributing to impaired glucose metabolism and insulin sensitivity within the liver.^[29]^ Insulin plays a crucial role in regulating TG levels, and excessive insulin secretion due to IR leads to hypertriglyceridemia, the most prevalent lipid abnormality in NAFLD.^[30]^ T2DM is closely associated with IR, leading to a significantly increased prevalence and severity of NAFLD among T2DM patients.^[31]^ Elevated fasting plasma glucose levels and ALT levels are indicators of increased NAFLD risk,^[32]^ with elevated ALT levels also associated with IR and predictive of T2DM development.^[33]^ The process of TG accumulation, known as lipotoxicity, damages pancreatic beta cells and exacerbates glucose homeostasis, particularly in hyperglycemic conditions.^[34]^ NAFLD and T2DM share common pathophysiological features, leading to the presumption of the occurrence of lipotoxicity and glucotoxicity. Therefore, the TyG index, a biomarker reflecting these characteristics, is considered excellent for predicting NAFLD and T2DM. In previous studies, the TyG index showed superior NAFLD and T2DM detection compared to HOMA-IR in both adult populations and children and adolescents.^[15,16]^

ALT has been used as a diagnostic tool for inflammation and liver damage and has been considered a surrogate marker for NAFLD.^[35,36]^ However, there is no consensus on the cutoff point and upper normal concentration of ALT. Furthermore, the thresholds of ALT values accepted previously may underdiagnose liver disease.^[37]^ Recognizing the clinical limitation of ALT, the SAFETY study, using data from the National Health and Nutrition Examination Survey, was conducted. This study reported that an ALT level greater than the 97th percentile was 26 U/L for men or 22 U/L for women, which are considered cutoff values suggesting liver disease in children and adolescents. NAFLD was diagnosed using the ALT criteria suggested by the SAFETY study. However, participants with hepatic steatosis also had normal ALT levels, and ALT levels could not distinguish between simple steatosis and NASH.^[38]^ Therefore, although ALT can help detect NAFLD, it alone may not be an ideal biomarker.

We hypothesized that a novel modified TyG index could further emphasize the characteristics of NAFLD by combining the TyG index, a surrogate marker of IR indicative of lipotoxicity and glucotoxicity, with ALT, an index associated with hepatocellular inflammation and injury.

Studies focused on modifying the TyG index by applying ALT to the TyG index are rare. In a cross-sectional study of an adult health checkup cohort, the TyG–ALT index obtained by multiplying the TyG index by ALT was superior to the TyG index in detecting NAFLD, with an AUC of 0.805.^[39]^ A study on the usefulness of the modified TyG index by applying ALT to the TyG index in children and adolescents has not been reported yet. This study presents a modified TyG index, the TyG–ALT index, which applies the pathophysiological characteristics of NAFLD, and demonstrates its usefulness for assessing the severity of NAFLD. The TyG–ALT index, calculated as ln(fasting TG [mg/dL] × fasting glucose [mg/dL] × ALT [U/L]/2), presented in this study is different from the formula presented in previous studies, and this is the first time that it has been related to the severity of NAFLD. However, since a healthy control group did not participate in this study, a cutoff value for the TyG–ALT index for detecting NAFLD could not be presented. To determine the ability of the TyG–ALT index to detect NAFLD, studies involving healthy controls without NAFLD are needed.

The TyG–ALT index presented in this study was found to be correlated with insulin and HOMA-IR, confirming that it has potential to be a surrogate marker of IR. The observation that both the TyG–ALT index and NAFLD severity increase with increasing US-FLI score is a novel finding that suggests both that the TyG–ALT index may reflect NAFLD severity and that US-FLI is associated with IR. Another important finding of this study is that the TyG–ALT index consistently reflected the increase in NAFLD severity as the US-FLI score increased. ALT was very good at detecting the severity of US-FLI ≥ 4 points, but its detection ability decreased as NAFLD severity increased. The TyG index had no detectable ability at US-FLI ≥ 4 points, but the detectable ability of the TyG index increased as the severity of NAFLD increased. In the case of the TyG–ALT index, an excellent detection ability was shown consistently in all sections from US-FLI ≥ 4 to ≥6 points (Table 5).

The limitations of this study are as follows. First, NAFLD diagnosis was based on ALT level measurement and ultrasound findings and was not confirmed by liver biopsy. However, it is considered valuable because NAFLD was diagnosed with US-FLI, which can represent liver biopsy results in patients with elevated ALT. In addition, US-FLI, a semiquantitative measurement tool, compensated for the subjective disadvantages of ultrasound and was highly correlated with the severity of NAFLD histopathology. Therefore, despite the lack of biopsy results, the results of this study are considered valuable. Second, because we analyzed cross-sectional data of only Korean children and adolescents, our results cannot be directly applicable to other races. Therefore, further research is needed in less homogeneous countries to determine the usefulness of the TyG–ALT index in different ethnic groups.

## 5. Conclusion

In conclusion, the TyG–ALT index is associated with the severity of US-FLI, suggesting that it can be used as a simple, inexpensive, and useful tool to predict the severity of NAFLD in clinical practice without liver biopsy or ultrasonography. Therefore, the TyG–ALT index is suggested as a useful biomarker that can be used to evaluate the severity of pediatric NAFLD in clinical practice.

## Author contributions

**Conceptualization:** Eu-Seon Noh, Il Tae Hwang.

**Data curation:** Eu-Seon Noh.

**Investigation:** Eu-Seon Noh.

**Writing – original draft:** Eu-Seon Noh.

**Writing – review & editing:** Eu-Seon Noh, Il Tae Hwang.
